# Validation of the Spanish version of the Edinburgh Feeding Evaluation in Dementia Scale for older people with dementia

**DOI:** 10.1371/journal.pone.0192690

**Published:** 2018-02-27

**Authors:** María Carmen Saucedo Figueredo, Juan Carlos Morilla Herrera, Mercedes San Alberto Giraldos, Inmaculada López Leiva, Álvaro León Campos, Celia Martí García, Silvia García Mayor, Shakira Kaknani Uttumchandani, José Miguel Morales Asencio

**Affiliations:** 1 Los Boliches Health Centre, Costa del Sol Primary Health Care District, Málaga, Spain; 2 Nursing Home Unit Málaga-Guadalhorce, Primary Health Care District, Málaga, Spain; Faculty of Health Sciences, University of Málaga, Málaga, Spain; 3 La Lobilla Health Centre, Costa del Sol Primary Health Care District, Málaga, Spain; 4 Department of Nursing, Faculty of Health Sciences University of Málaga, Málaga, Spain; 5 Department of Nursing, Comillas University, Salamanca, Spain; 6 Red Cross School of Nursing, Sevilla, Spain; Taipei Veterans General Hospital, TAIWAN

## Abstract

**Aims:**

To adapt the Edinburgh Feeding Evaluation in Dementia Scale (EdFED) for use in a Spanish-speaking population and to assess its validity and reliability in patients with dementia.

**Method:**

A cross-sectional study was carried out in two stages: 1. Cross-cultural adaptation (translation, back-translation, review by committee of experts, pilot test and weighting of results); 2. Clinimetric validation comprising interobserver reliability assessment, test-retest reliability and internal consistency. To determine construct validity, confirmatory factorial analysis and principal components analysis were performed by oblique rotations. Criteria validity was analysed using the Pearson correlation (p<0.05) with the BMI, MNA and analytical values of albumin, transferrin, cholesterol, absolute lymphocytes and total proteins.

Data collection was carried out for six months in 2016 in nursing homes and Alzheimer’s day centers in the province of Málaga (Spain), at nine centers, with 262 patients (aged over 60 years and presenting feeding difficulties), 20 nurses, 20 professional caregivers and 103 family caregivers.

**Results:**

A version of EdFED culturally adapted to Spanish was obtained. The sample presented the following characteristics: 76.3% women, mean age 82.3 years (SD: 7.9); MNA 18.73 (SD: 4.44); BMI 23.99 (SD: 4.72); serum albumin 3.79 mg/dl (SD: 0.36). A Cronbach’s alpha of 0.88 was obtained, with an inter-item global correlation of 0.43 and a homogeneity index ranging from 0.42 to 0.73. The exploratory factor analysis reproduced the three-factor model identified by the original authors, explaining 62.32% of the total variance. The criterion validity showed a good inverse correlation with MNA and a moderate one with albumin, total proteins, transferrin and BMI.

**Discussion:**

The Spanish version of EdFED is reliable and valid for use in elderly people with dementia. The most appropriate for our environment is the three-factor model, which maintains the original factors, with a slight redistribution of the items.

## 1. Introduction

By 2050, the number of people aged over 65 years will have tripled [1,2], and Spain will be one of the oldest countries in the world [3]. In consequence, due to the close association between dementia and aging, it is estimated that the number of people affected by this condition will also have risen three-fold [4,5]. One of the problems commonly associated with dementia is the appearance of undernourishment, which is estimated to be present in 5–8% of persons in the community, in 13–60% of those in nursing homes and in 37–39% of those in hospitals [6–9].

A further problem is that up to 40% of people with dementia are already undernourished when the condition is diagnosed [10], and this tends to worsen during their illness, with negative consequences for their progress [1]. In the early stages of dementia, it affects instrumental abilities (causing difficulty in doing the shopping, cooking, etc.), while the moderate stage is characterised by loss of appetite, behavioural disorders and apraxia (loss of ability to perform a movement). In the advanced stages, dependence becomes more visible and acute, and can lead to difficulty in swallowing [11,12]. Feeding difficulties affect 70% of patients in the advanced stages of dementia [12–15]. “Difficulty in feeding” is taken to mean any condition that causes a reduction in food intake [16], affecting any of five areas: initiating feeding, maintaining attention, taking or keeping food in the mouth, chewing and swallowing [17]. Other difficulties in feeding are the inability to recognise or use utensils; recognising food for what it is; difficulty in opening the mouth, in placing food in the mouth, in keeping food within the mouth and/or in closing the mouth; turning the head away; spitting out food; leaving the mouth open and allowing food to fall; not swallowing food; playing with it; or making gestures of discomfort/pain [18]. The first difficulty in feeding is usually “refusing to eat” and one of the last, indicating severity, is “leaving the mouth open” [19].

It is important for difficulties in feeding to be identified at an early stage, in order to establish effective interventions to prevent complications, improve nutritional status and overall health and thus decrease the high morbidity and mortality observed in these patients. One of the most widely-used approaches to this challenge is the creation and use of instruments to assess feeding difficulties in patients with dementia [16,20–35]. However, many such instruments were developed in the last century and are not always directly aimed at evaluating this problem. Moreover, the majority are used in cases of acute hospitalisation, while others have not been implemented in practice or have been evaluated by non-robust methods.

Of all of these instruments, the EdFED presents the highest validity and reliability [36,37]. It was developed to be applied by nurses to evaluate feeding difficulties in patients with moderate-advanced dementia in long-stay centers. EdFED has high internal consistency (Cronbach’s alpha 0.87) and optimal Mokken scalability (H^t^ coefficient = 0.27) [38,39]. It consists of 11 questions or items: the first four address the level of care required by the patient; the next six concern behaviours that reflect the patient’s deterioration; and the last item determines the level of care required: supervision or light help, partial help or total help. Each question has three possible answers, which are scored from 0 to 2, according to how many times the behaviour in question is observed (never, sometimes or often), to define how these terms are to be quantified, it is considered for this study that "sometimes" is when you observe the behavior 2 or 3 times and "often" is when you observe more than 4 times in the meals of the last week. The participants were instructed in this way. The score pattern for this instrument is cumulative and hierarchical, to represent the progressive worsening of the patient’s behaviour. The original version was designed to be completed by observing the patient, but its validity was subsequently verified by adding information provided verbally by the caregiver and/or data from the patient’s medical history. Accordingly, the presence of the patient or the nurse was no longer required [37,40,41]. EdFED has been adapted and validated in other languages (Chinese and Italian) [19,42–45], but to date no Spanish version has been made available. Furthermore, in Spain there is no similar tool that has been adapted or validated, and which is specific for elderly people with dementia. Accordingly, most health professionals use instruments aimed at evaluating nutritional status, but not difficulties in feeding.

The aim of this study is to obtain a version of EdFED adapted transculturally to the Spanish context for use in institutionalised and community-based dementia patients, and to empirically evaluate its clinimetric properties and reliability when administered by different providers (nurses, professional caregivers or family caregivers).

## 2. Methods

### 2.1. Design

A two-stage clinimetric validation study was carried out: in the first stage, EdFED was cross-culturally adapted to the Spanish context, and in the second it was empirically validated for use by nurses, professional caregivers and family caregivers.

### 2.2. Population and sample

The study was carried out in nine nursing homes and community day-care centers for people with dementia in Málaga (Southern Spain). The subjects were all older than 60 years, institutionalised for at least three months, diagnosed with dementia and required feeding assistance (assessed with the feeding assistance dimension of the Barthel index, with a score ≤5). People who were terminally ill, or who presented with additional illnesses that impeded or prevented feeding (stroke, amyotrophic lateral sclerosis, motor neuron disease, Parkinson’s, fractures, paralysis) were excluded, as were persons requiring nasogastric, gastro- or jejunostomy tube feeding or artificial nutrition of any type or who did not provide consent to participate.

For the empirical validation phase, to test the hypotheses of the two, three and four-factor structural models previously identified in the literature, assuming a RMSEA of 0.05, an alpha value of 0.05 and a maximum of 66 degrees of freedom, it was calculated that 175 subjects were required. Given the characteristics of the study population, this sample size was increased by 50% to cover possible losses and to be able to perform subgroup analyses if necessary. All calculations were performed using Statistica 12 software [46]

In the cultural adaptation stage, the following steps were followed, as proposed by the International Test Commission [47] and by Guillemin, Bombardier, and Beaton (1993) [48]: translation, back-translation, and review by a committee of experts, weighting of results, and a final pilot test. Prior authorization for this study was obtained from the original author of the instrument.

The scale was translated from English into Spanish by six professional translators, whose mother tongue was Spanish, working independently. Three of them were aware of the purpose of the instrument and the concepts involved, while the other three performed the translation without that knowledge. This procedure facilitated the simultaneous literary and conceptual translation. Translations to the questions 8, 10 and 11 were the most coincident. The rest were very similar (did not lose meaning in essence) except for the use of pronouns or word placed.

A panel of 20 experts, working in accordance with the Moriyama criteria [49], then reviewed the different 6 versions produced, to assess whether the components were clearly defined, the feasibility of obtaining useful information from the answers given to each question, the comprehensibility of each item, the possible redundancy of some of them, and the a priori capacity of the different items to discriminate between degrees of feeding difficulty. Finally, the panel evaluated the semantic and cultural equivalence achieved. Each of these aspects was evaluated by each expert on a Likert scale ranging from 1 to 9. Agreement was considered to have been reached when the percentile of scores was higher than 75 (median equal or over 7) and when the dispersion of scores did not exceed an interquartile range of 3. Medians and the RICs for each proposed translation were scored, the consensus was quite good, 100% obtained medians over 7, only questions 5 and 8 needed a second round.

The final version in Spanish was then back-translated into the original language by three native English-language translators, who had not participated in the first stage and who worked independently of each other. The resulting translations were compared to the original scale for possible inconsistencies.

In order to verify the instrument’s comprehensibility to its end users, ten patients at nursing homes and Alzheimer’s day centers were given a copy of the questionnaire by nurses, professional caregivers or relatives, and asked in cognitive interviews to identify the meaning of each item. No difficulties were detected in this regard in any case.

### 2.3. Data collection

Data collection was carried out over six months (May-September 2016), with the participation of 20 nurses, 20 professional caregivers and 103 family caregivers. For the patients with Dementia at Alzheimer’s day centre and those who were living in the community, the three observers (nurse, professional caregiver and family caregiver) responded to the EdFED questionnaire on the same patient, providing three different views. All the participants were trained to use the instrument during a face-to face session. Data were collected on three different meals for the same patient: the breakfasts and lunches provided at the centre, and dinners, which were eaten at home. In the case of the (institutionalised) patients in nursing homes, only the nurse and the professional caregiver, being familiar with the patient’s eating habits, were in a position to answer the questionnaire, since in most cases the family caregivers were unknown to the researchers, or were not present, or did not usually participate in feeding the patient at the centre. Thus, two outlooks were obtained for each patient and for each meal, since both the nurses and the professional caregivers helped/supervised the patient’s feeding.

The following data were collected for each patient: age, sex, functional status (according to the Barthel index), cognitive status (Pfeiffer test), nutritional status (Mini Nutritional Assessment—MNA), forearm circumference (FC), calf circumference (CC), weight, height (for patients who could not be weighed or measured, because they were bedridden or suffered problems in one or more limbs, alternative, validated anthropometric markers were obtained, using parameters such as forearm length, heel-knee distance, FC or CC) [50–52], BMI, biochemical and immunological markers of undernourishment, obtained by blood tests (albumin, transferrin, absolute lymphocytes, cholesterol and total proteins). In addition, the age, sex and relationship of the primary family caregiver were determined.

### 2.4. Ethical issues

Prior approval for the study was obtained from the Costa del Sol Ethics and Research Committee (on 1 December 2014) and for the corresponding body for the province of Málaga (on 30 November 2015). In addition, the management bodies of each health district involved, and of the participating centers, all gave their approval. All subjects were asked for their consent to participate in the study; in cases of cognitive impairment, permission was sought of their legal representative or guardian. The capacity to consent was determined by Pfeiffer test. If a value higher than four was obtained, permission was requested to their representatives.

### 2.5. Analysis

Descriptive statistics of the variables were obtained, and the normality of their distribution was confirmed by the Kolmogorov-Smirnov test. Bivariate analysis was performed using Student’s t test, the chi-square test, Wilcoxon’s test, the Mann-Whitney U test and Pearson’s or Spearman’s correlations, according to the normality or otherwise of the distributions and the type of variables being considered. When non-homoscedasticity (according to the Levene test) was observed, ANOVA was used with measures of central robustness, applying the Welch and the Brown-Forsythe tests. All these calculations were performed with 95% confidence intervals (95%CI).

For the clinimetric validation, interobserver reliability and test-retest reliability (between information provided by nurses and caregivers about the same patient for different meals or about the same meal) were evaluated using Pearson correlation coefficients and intraclass correlation coefficients. Internal consistency was also assessed, by Cronbach’s alpha.

For construct validity, confirmatory factorial analysis was carried out to verify the validity of previous two, three and four-factor proposals in this field of research. The goodness of fit of the models was assessed by the following indexes: penalty function (χ2/df), which indicates a good fit with values <3; the root mean square error of approximation (RMSEA index) and its 90% confidence interval, taking 0.05 as a cut-off value for good fit; the normed fit index (NFI); the comparative fit index (CFI); the goodness of fit index (GFI), with a range of 0–1 and for which the minimum value of good fit is 0.90; and the standardized root mean square residual (SRMR) index, which indicates a good fit with values <0.08. Bartlett’s sphericity test and the KMO test were performed to determine the relevance of the factorial analysis. The ceiling/floor effect of each item was estimated by the endorsement frequency, with a limit value of 20%.

The criterion validity was analysed by comparing the EdFED scores obtained with the corresponding values for albumin, transferrin, cholesterol, total lymphocytes, total protein, BMI and MNA.

## 3. Results

The content validation and cultural adaptation stage produced a broad consensus, and agreement failed to be reached on only two questions, Nos. 5 and 8, which had to be submitted to a second round of consideration, after which the final Spanish-language version of the scale was obtained (S1 File).

The empirical sample presented the following characteristics: 262 subjects were recruited, of whom 76.3% were women; the mean age of the patients was 82.3 years (SD: 7.9); 39% attended day centers and 61% were resident in nursing homes. 78% of the family caregivers were women and in over half of the cases they were caring for their own parents.

In the community, both nurses and formal caregivers observed an average of 4.68 patients per meal (breakfast and lunch), and in nursing homes the average of both nurses and formal caregivers was 5.46 patients per meal (lunch). Informal caregivers observed only their relative in a single meal (dinner).

The patients had high levels of functional (82%) and cognitive (65%) dependence, as shown in detail in Table 1.

**Table 1:** Functional status of patients.

	Total: [n = 262] n (%)	Male [n = 62; 23.7%] n (%)	Female [n = 200; 76.3%] n (%)	p	Nursing homes [n = 159; 60.7%] n (%)	Community [n = 103; 39.2%] n (%)	p
**Global functionality (Barthel Index)**							
Severe dependence	210 (80.2)	43 (69.4)	167 (83.5)	0.024	143 (89.9)	67 (65.0)	<0.001
Moderate dependence	52 (19.8)	19 (30.6)	33 (16.5)		16 (10.1)	36 (35.0)	
**Feeding functionality (Barthel)**							
Dependent	109 (41.6)	22 (35.5)	87 (43.5)	0.303	81 (50.9)	28 (27.2)	<0.001
Needs help cutting, spreading butter, etc., or requires modified diet	153 (58.4)	40 (64.5)	113 (56.5)		78 (49.12)	75 (72.8)	
**Cognitive Status (Pfeiffer)**							
Severe impairment	170 (64.9)	33 (53.2)	137 (68.5)	0.046	119 (74.8)	51 (49.5)	<0.001
Moderate impairment	65 (24.8)	18 (29.0)	47 (23.5)		33 (20.8)	32 (31.1)	
Slight impairment	14 (5.3)	7 (11.3)	7 (3.5)		3 (1.9)	11 (10.7)	
Normal	13 (5.0)	4 (6.5)	9 (4.5)		4 (2.5)	9 (8.7)	

Regarding the patients’ anthropometric measurements and nutritional status, the majority presented BMI values within the normal range, although moderate degrees of undernourishment were detected in 17% of patients, and according to the MNA criteria, half of the sample were at risk of malnutrition (Table 2). Energy and protein reserves, assessed by FC and CC respectively, were within normal limits in most cases, but reflected risk of undernourishment in 23% of cases according to FC and in 39% according to CC (Table 2).

**Table 2:** Anthropometric parameters, nutritional status and energy reserve measures.

	Total: [n = 261] n (%)	Male [n = 61; 23.4%] n (%)	Female [n = 200; 76.6%] n (%)	p	Nursing homes [n = 159; 60.5%] n (%)	Community [n = 103; 39.5 n (%)	p
**Anthropometric parameters, nutritional status and energy reserve measures (BMI)**							
Severe undernourishment <19	36 (13.8)	3 (4.9)	33 (16.5)		32 (20.1)	4 (3.9)	<0.001
Moderate undernourishment 19–21	44 (16.9)	9 (14.8)	35 (17.5)		33 (20.8)	11 (10.8)	
Slight undernourishment 21–23	32 (12.3)	8 (13.1)	24 (12.0)		21 (13.2)	11 (10.8)	
Normal weight (>23)	149 (57.0)	41 (67.2)	108 (54.0)		73 (45.9)	76 (74.5)	
**Energy and protein reserves (FC/CC)**							
**Forearm circumference (FC)**							
Undernourishment<21	24 (9.2)	2 (3.3)	22 (11.0)	0.080	22 (13.8)	2 (2.0)	0.001
Risk of undernourishment (21–22)	59 (22.6)	11 (18.0)	48 (24.0)		48 (30.2)	11 (10.8)	
Normal >22	178 (68.2)	48 (78.7)	130 (65.0)		89 (56.0)	89 (87.2)	
**Calf circumference (CC)**							
Undernourishment< 31	102 (39.1)	17 (27.9)	85 (42.5)	0.057	72 (45.3)	30 (29.4)	0.015
Normal (≥31)	159 (60.9)	44 (72.1)	115 (57.5)		87 (54.7)	72 (70.6)	
**Nutritional assessment (MNA)**							
Malnutrition <17	91 (34.9)	19 (31.1)	72 (36.0)	0.274	60 (43.4)	22 (21.6)	0.001
Risk of malnutrition 17–23.5	131 (50.2)	29 (47.6)	102 (51.0)		82 (51.6)	49 (48.0)	
Normal state of nutrition >23.5	39 (14.9)	13 (21.3)	26 (13.0)		8 (5.0)	31 (30.4)	

### 3.1. Reliability and consistency

The instrument obtained excellent internal consistency. The Cronbach’s alpha values were 0.88 for the version used by nurses, 0.97 for professional caregivers and 0.86 for family caregivers.

The inter-item correlation matrices and indexes of homogeneity are presented in detail in Table 3. For inter-observer reliability, the intra-class correlation coefficient between the nurses and professional caregivers’ scores was 0.94 (p<0.0001); between nurses and family caregivers, it was 0.81 (p<0.0001); and between professional caregivers and family caregivers it was 0.78 (p<0.0001). No ceiling/floor effect was observed for any item or for any observer.

**Table 3:** Index of homogeneity of the scale, as administered by nurses and by professional caregivers n = 262.

**Nurses**					
	Mean value when the item is removed	Variance when the item is removed	Corrected item-total correlation	Multiple correlation squared	Cronbach’s alpha when the item is removed
EdFED1	5.50	17.814	.673	.648	.871
EdFED2	5.85	17.210	.726	.779	.868
EdFED3	6.15	18.898	.573	.458	.878
EdFED4	6.04	19.565	.422	.370	.888
EdFED5	6.31	19.554	.548	.509	.879
EdFED6	6.56	19.358	.653	.576	.874
EdFED7	6.58	19.402	.673	.633	.873
EdFED8	6.61	19.511	.656	.585	.874
EdFED9	6.72	20.280	.544	.499	.880
EdFED10	6.68	20.205	.584	.501	.879
EdFED11	5.91	16.888	.732	.744	.868
**Certified Nurse Assistants**					
	Mean value when the item is removed	Variance when the item is removed	Corrected item-total correlation	Multiple correlation squared	Cronbach’s alpha when the item is removed
EdFED1	5.88	18.078	.665	.681	.857
EdFED2	6.19	17.480	.736	.805	.851
EdFED3	6.43	19.317	.549	.381	.865
EdFED4	6.26	21.237	.237	.229	.887
EdFED5	6.60	19.983	.561	.467	.864
EdFED6	6.80	19.595	.649	.462	.859
EdFED7	6.81	19.593	.691	.611	.857
EdFED8	6.89	20.191	.588	.428	.863
EdFED9	7.01	20.909	.536	.421	.867
EdFED10	6.95	20.612	.584	.513	.865
EdFED11	6.25	17.432	.720	.748	.852

### 3.2. Construct validity

**Fig 1:**
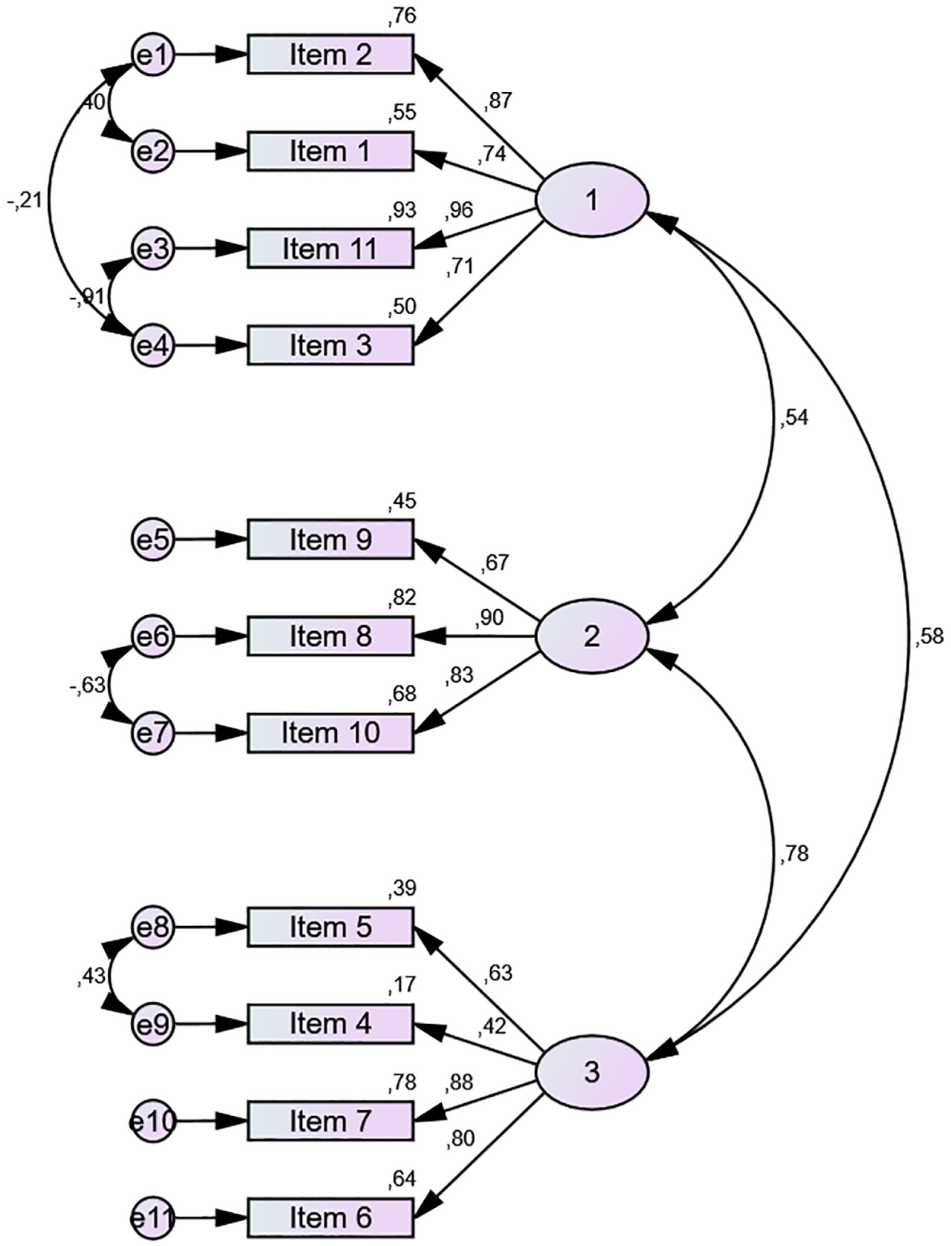
Factor structure of the Spanish version of EdFED.

The KMO and Bartlett’s sphericity tests showed appropriate values for factorial analysis to be performed. The confirmatory analysis (CFA) of the two-factor structure of the original model; ‘Factor 1: patient obstinacy or passivity’ and ‘Factor 2: nursing interventions’, explained 72.2% of the variance, although the goodness-of-fit parameters were only modest. The three-factor structure of the original model (Factor 3 is named: ‘indicator of feeding difficulty’), explained 62.4% of the variance, with poor goodness-of-fit indexes. An additional three-factor structure was tested using an exploratory factorial analysis performed with a subsample that was later verified by CFA in the remaining sample; this explained 62.3% of the variance and provided better goodness-of-fit parameters than the original three-factor structure (Fig 1).

Finally, the test of the four-factor structure (Factor 4 is named “oral aspects or feeding difficulty”) offered very poor goodness-of-fit parameters. Table 4 shows the detailed results of each goodness-of-fit index, for all models.

**Table 4:** Goodness-of-fit parameters of the CFA for all models considered, n = 262.

	CMIN/DF	GFI	NFI	CFI	RMSEA	90%CI
Original 2-factor	3.04	0.93	0.93	0.95	0.09	0.07 to 0.10
Original 3-factor	3.17	0.92	0.92	0.94	0.10	0.08 to 0.12
Spain 3-factor*	2.71	0.94	0.94	0.96	0.08	0.06 to 0.10
Original 4-factor	3.28	0.93	0.93	0.95	0.09	0.07 to 0.11

*Selected

Bifactorial: Factor 1 (Questions: 1, 2, 4, 5, 6, 7, 8, 9, 10), Factor 2 (Questions 1, 2, 3, 11)

Trifactorial: Factor 1 (Questions: 6, 7, 8, 9), Factor 2 (Questions: 1, 2, 3, 11), Factor 3 (Questions: 4, 5)

Tetrafactorial: Factor 1 (Questions: 6, 7, 8, 10, 11), Factor 2 (Questions: 1, 2, 3), Factor 3 (Questions: 4, 5), Factor 4 (Questions: 8, 9, 10)

EdFED scores were significantly different between elders admitted in institutions and those living in the community, being higher in the first group (Table 5).

**Table 5:** Differences in EdFED scores between institutionalized and community subjects.

	Institutionalized (n = 159)	Community (n = 103)	
	Mean (SD)	Mean (SD)	p
EdFED Nurses	7,72 (4,36)	5,60 (5,08)	<0,0001
EdFED professional caregivers	7,98 (4,60)	5,72 (4,92)	<0,0001
EdFED caregivers	NA	6,39 (4,50)	-

### 3.3. Criterion validity

Complete blood samples were obtained from 196 subjects (drop-out 25.2%).

There was a moderately-significant inverse correlation with BMI values and a significant one with MNA. For the EdFED scores and the analytical parameters, the only correlation observed was a significant, inverse but very slight one with total serum proteins, serum albumin and transferrin. There was no relation with total cholesterol or with the lymphocyte count (Table 6).

**Table 6:** Correlation between EdFED (Nurses) and BMI, MNA and biochemical parameters n = 196.

	Correlation coefficient*	p
BMI	-0.30	<0.001
MNA	-0.62	<0.001
Albumin	-0.279	<0.001
Lymphocytes	0.106	0.143
Cholesterol	0.021	0.772
Total protein	-0.151	0.036
Transferrin	-0.298	<0.001

BMI: Body Mass Index, MNA: Mini Nutritional Assessment

*Spearman’s rho

## 4. Discussion

The aim of this study was to obtain a valid, reliable version of EdFED adapted culturally to the Spanish context, to assess the feeding difficulties encountered by elderly persons with dementia. Our results show that the version obtained is sufficiently valid and reliable for use in the cultural context of Spain, with a three-factor construct that differs slightly from the original model, which also contained three factors: patient obstinacy or passivity, nursing intervention and indicator of feeding difficulty. Behaviours while eating or being feed are explained by these 3 factors where test questions are grouped [36]. Depending on the factor affected nurses can organize care with the most appropriate intervention. These factors help nurses organize nursing care and address them with concrete interventions. In this model, the first factor contains the items or questions 6–10; the second, numbers 1–3, and 11; and the third, numbers 4 and 5. In the three-factor model obtained in our study, items 6 and 7, related to the obstinacy/passivity factor in the Watson model, would be included in the ‘level of difficulty’ factor. Indeed, in both the original version and the model presented here, it is hard to discern, conceptually, whether items such as “the patient sometimes refuses to eat” or “refuses to open his/her mouth” correspond more to passivity or to obstinacy behaviours, or to situations of feeding difficulty. Both approaches are plausible, which explains why in both versions there are fluctuations between factors in items that could equally well be in a single, common factor, although two-factor models do not achieve a satisfactory goodness of fit in our study or in that of [36]

However, as with any clinimetric instrument, subsequent assessments in different contexts will ultimately determine the factorial behaviour of the instrument. It should not be forgotten that the clinimetric validation process implies a continual succession of empirical confirmations of the factorial structure, in different contexts and populations, conducted to obtain cumulative evidence of the clinimetric properties of the instrument [53]

A notable finding is that item 4 presented a poorer psychometric behaviour than all other items. This may be related to the fact that this item does not measure uneaten food or the amount of nutritional intake in 24 hours (unlike the dish method), but considers behaviours/attitudes during meals. It would be interesting to test a version in which this item were replaced with one based on an analysis of food left on the plate, and to determine its influence on the overall performance of the scale.

No previous study has analysed the relationship between feeding difficulties and analytical markers of nutritional status. Our criterion validity test for these analytical parameters showed that higher values of the EdFED scale are associated with lower ones for parameters such as albumin, total proteins and transferrin. In other words, the greater the alteration of the patient’s feeding behaviour, the stronger the evidence of undernourishment provided by these biochemical markers. The use of these parameters, however, has been strongly criticized because they are easily altered by other organic problems [54,55]. However, studies have shown that these markers present sufficient sensitivity and specificity to assess nutritional fluctuations in an elderly population [56]. Moreover, some authors believe them to be a useful tool for non-diagnostic screening, because undernourishment, regardless of its cause, provokes tissue and cellular alterations that are immediately and continuously recognisable with these markers [57].

The inverse correlation between EdFED and MNA shows there is a relationship between a greater number of altered behaviours and the patient’s undernourishment. With respect to BMI, a higher percentage of body mass is also correlated inversely with EdFED, indicating that an appropriate body weight is associated with fewer difficulties in feeding. The extent to which feeding problems might act as predictors of the risk of undernourishment or of malnutrition is a question that should be addressed in future studies, with longitudinal prospective designs. This could represent an important area of advance in the care for such patients, and point the way to designing early-action initiatives to prevent nutritional disorders.

This paper could be potentially construed to draw a predictive association between the EdFED and malnutrition. However, this study was not powered to test this association. Neither can one say that malnutrition as defined by the MNA and the other biomarkers predicts dementia and further studies in the future could confirm these findings.

The results of our study not only support its use in long-term nursing homes, but also in the community context, a different cultural environment from that for which it was designed, but one in which mechanisms are needed for evaluating feeding difficulties, since it is in this environment where such patients are mostly found [58,59]. The profile of the patients included in our study is very similar to that usually found in nursing-home and community settings [8], which are a relevant consideration, as persons with dementia cannot always be assessed by a nurse when feeding, as this possibility depends on the context of care. The instrument we describe can easily be used by nurses in settings such as domestic or nursing-home care, without their presence being necessary, but making use of the valuable clinical information it provides, in order to make decisions in planning the patient’s care.

EdFED is one of the few instruments currently available to assess feeding difficulties in conditions of validity and reliability, specifically designed for elderly persons with moderate-advanced dementia. This study also verifies the reliability of the instrument for use not only by nurses, but also by family caregivers and professional caregivers; this approach is novel, and the information had not been obtained in previous studies [16,19,20,43–45,60].

The availability of a Spanish-language version of EdFED opens up a new way of assessing feeding difficulties experienced by elderly patients with dementias, and suggests a pathway towards early-action solutions other than standard practice in this situation, such as nutritional supplements or enteral nutrition, policies that are usually undertaken when nutritional problems are already apparent. Further studies are needed to verify whether an intervention based on this type of assessment would influence the type of measures implemented with these patients.

Our study has the limitation that it does not differentiate between the types of dementia that may be presented, in the view that this might bias the conclusions drawn, due to the large differences found in the evolution of different types of dementia. Furthermore, there is great complexity in placing a diagnostic label on dementia, because of the difficulty encountered by these elderly and impaired patients in gaining access to a specialist consultation. In consequence, the categories assigned in the corresponding medical records are frequently incorrect. It could be possible that those subjects institutionalized under six months, might still have levels of translocation syndrome, which might result in refusing to eat.

The observation in the dinners was different with respect to breakfasts and lunches, since in these last cases, the observers evaluated several participants, while in the dinners, being in the homes, the observation was more individualized and perhaps this could affect to interobserver reliability.

Finally, in this study it was not possible to evaluate test-retest reliability or the sensitivity to change, due to the characteristics of the sample collection process and the study design. Obtaining this information would require longitudinal follow-ups, at appropriate intervals, to evaluate each of these aspects.

## Supporting information

S1 File Spanish version of EdFED. (DOCX)

S2 File Database. (CSV)
