# Supplementary material for: Validation of the Spanish version of the Edinburgh Feeding Evaluation in Dementia Scale for older people with dementia
Source: PLoS One. 2018 Feb 27;13(2):e0192690. doi: 10.1371/journal.pone.0192690 (PMC5828442; doi:10.1371/journal.pone.0192690)
Supplement: S1 File — (DOCX) [file pone.0192690.s001.docx]

**S1. Supplement 1. Spanish version of EdFED**

1. ¿Requiere el paciente estrecha supervisión durante la alimentación?
2. ¿Requiere el paciente ayuda física con la alimentación?
3. ¿Se le derrama la comida mientras se alimenta?
4. ¿Tiende el paciente a dejar comida en el plato al final de la comida?
5. ¿Se niega el paciente alguna vez a comer?
6. ¿Vuelve el paciente la cabeza hacia otro lado mientras se le alimenta?
7. ¿Se niega el paciente a abrir la boca?
8. ¿Escupe el paciente su comida?
9. ¿Deja el paciente la boca abirta permitiendo que la comida se caiga fuera?
10. ¿Se niega el paciente a tragar?

Cada preguntas tiene tres posibles respuestas:

Nunca (0 puntos)

A veces (1 punto)

Casi Siempre (2 puntos)

1. Indique el nivel adecuado de cuidado para la alimentación que require el paciente:
   1. Apoyo-Educativo
   2. Parcialmente compensatorio
   3. Totalmente compensatorio
